# GII.13/21 Noroviruses Recognize Glycans with a Terminal β-Galactose via an Unconventional Glycan Binding Site

**DOI:** 10.1128/JVI.00723-19

**Published:** 2019-07-17

**Authors:** Xin Cong, Xiao-man Sun, Jian-xun Qi, Han-bo Li, Wen-gang Chai, Qing Zhang, Hong Wang, Xiang-yu Kong, Jiao Song, Li-li Pang, Miao Jin, Dan-di Li, Ming Tan, Zhao-jun Duan

**Affiliations:** a Key Laboratory for Medical Virology and Viral Diseases, National Health Commission of the People’s Republic of China, Beijing, China; b National Institute for Viral Disease Control and Prevention, China CDC, Beijing, China; c Institute of Microbiology, Chinese Academy of Sciences, Beijing, China; d School of Public Health, Gansu University of Traditional Chinese Medicine, Lanzhou, China; e Glycosciences Laboratory, Department of Medicine, Imperial College London, London, United Kingdom; f Division of Infectious Diseases, Cincinnati Children’s Hospital Medical Center, Cincinnati, Ohio, USA; g Department of Pediatrics, University of Cincinnati College of Medicine, Cincinnati, Ohio, USA; Instituto de Biotecnologia/UNAM

**Keywords:** crystal structure, glycan, histo-blood group antigens, human norovirus, viral receptor

## Abstract

Human noroviruses (huNoVs) recognize histo-blood group antigens (HBGAs) as host susceptibility factors. GII.13 and GII.21 huNoVs form a unique genetic lineage that emerged from mainstream GII NoVs via development of a new, nonconventional glycan binding site (GBS) that binds Le^a^ antigen. This previous finding raised the question of whether the new GII.13/21 GBS really has such a narrow glycan binding spectrum. In this study, we provide solid phenotypic and structural evidence indicating that this new GBS recognizes a group of glycans with a common terminal β-galactose (β-Gal). First, we found that P domain proteins of GII.13/21 huNoVs circulating at different times bound three glycans sharing a common terminal β-Gal, including Lec, lactose, and mucin core 2. Second, we solved the crystal structures of the GII.13 P dimers in complex with Lec and mucin core 2, which showed that β-Gal is the major binding saccharide. Third, nonfat milk and lactose blocked the GII.13/21 P domain-glycan binding, which may explain the low prevalence of GII.13/21 viruses. Our data provide new insight into the host interactions and epidemiology of huNoVs, which would help in the control and prevention of NoV-associated diseases.

**IMPORTANCE** Evidence from both phenotypic binding assay and structural study support the observed interactions of human noroviruses (huNoVs) with histo-blood group antigens (HBGAs) as receptors or attachment factors, affecting their host susceptibility. GII.13 and GII.21 genotypes form a unique genetic lineage that differs from the mainstream GII huNoVs in their unconventional glycan binding site. Unlike the previous findings that GII.13/21 genotypes recognize only Le^a^ antigen, we found in this study that they can interact with a group of glycans with a common terminal β-Gal, including Lec, lactose, and mucin core 2. However, this wide glycan binding spectrum in a unique binding mode of the GII.13/21 huNoVs appears not to increase their prevalence, probably due to the existence of decoy glycan receptors in human gastrointestinal tract limiting their infection. Our findings shed light on the host interaction and epidemiology of huNoVs, which would impact the strategy of huNoV control and prevention.

## Abstract (precis)

Evidence from both phenotypic binding assay and structural study support the observed interactions of human noroviruses (huNoVs) with histo-blood group antigens (HBGAs) as receptors or attachment factors, affecting their host susceptibility. GII.13 and GII.21 genotypes form a unique genetic lineage that differs from the mainstream GII huNoVs in their unconventional glycan binding site. Unlike the previous findings that GII.13/21 genotypes recognize only Le^a^ antigen, we found in this study that they can interact with a group of glycans with a common terminal β-Gal, including Lec, lactose, and mucin core 2. However, this wide glycan binding spectrum in a unique binding mode of the GII.13/21 huNoVs appears not to increase their prevalence, probably due to the existence of decoy glycan receptors in human gastrointestinal tract limiting their infection. Our findings shed light on the host interaction and epidemiology of huNoVs, which would impact the strategy of huNoV control and prevention.

## INTRODUCTION

Human noroviruses (huNoVs), members of the *Norovirus* genus of the *Caliciviridae* family, are the leading cause of acute gastroenteritis (AGE) and are associated with almost 20% of all AGE cases worldwide (1). NoVs are RNA viruses that contain a single-stranded, positive-sense RNA genome consisting of three open reading frames (ORFs) (2). ORF1 encodes six nonstructural (NS) proteins responsible for viral genome replication, while ORF2 and ORF3 encode major and minor capsid proteins. The major capsid protein (VP1) is composed of a shell (S) domain and a protruding (P) domain, and the P domain is further divided into the P1 and P2 subdomains. The P2 subdomain is the main determinant of NoV diversity, antigenicity, and glycan binding patterns.

NoVs are classified into seven genogroups (GI to GVII), among which GI, GII, and GIV cause human infections (1). HuNoVs recognize histo-blood group antigens (HBGAs) as receptors or attachment factors that are believed to be important for huNoV infection and host susceptibility (3–6). HBGAs are fucose-containing glycans and the determinants of various blood types, including ABO, Lewis, and secretor status. While the HBGAs are polymorphic and diverse in interacting with different huNoVs, GI and GII huNoVs showed genogroup-specific binding modes (6). Specifically, the majority of GII huNoVs recognize the α-fucose (α-Fuc) of HBGAs as the major binding saccharide (MaBS), while GI huNoVs interact with the β-galactose (β-Gal) as the MaBS (5). In all these cases, huNoVs also interact with at least another saccharide to support or stabilized the binding outcomes.

In addition to HBGAs, huNoVs can also recognize other glycans or small chemicals. For example, some huNoVs (GI.3 VA115 and GII.4 VA387) reportedly bind gangliosides and sialic acid-containing glycoconjugates (7), similar to murine norovirus (MNoV) (8) and feline calicivirus (9). On the other hand, animal caliciviruses are reported to recognize carbohydrates as attachment factors; for example, bovine NoV binds to α-Gal (10) and canine and bat NoVs recognize the A and H antigens (11, 12). Also, some huNoVs and MNoVs bind bile acids as cofactors, which may enhance huNoV-HBGA interactions (13). Moreover, human milk oligosaccharides (HMOs) act as natural decoys for huNoVs, which may reduce the risk of huNoV infection of milk-fed infants (14–16). The above scenarios indicate the complexity and diversity of huNoV-glycan interactions.

The HBGA binding profiles of huNoVs change over time within a genotype, which may contribute to their prevalence. For example, a new variant of the previously rare genotype GII.17 became the predominant strain to cause outbreaks during the epidemic season of 2014–2015 (17, 18). Along with the antigenic change (17), the new GII.17 variant had a broader HBGA binding spectrum due to minor mutations at the conserved GII glycan binding site (GBS) (18–21). Therefore, identification of the new huNoV-glycan interactions, especially those of the rare genotypes, is necessary to help in the prevention and control of huNoV-associated diseases. Strikingly, unlike the mainstream GII huNoVs, which share a highly conserved GBS that binds various combinations of HBGAs, GII.13 and GII.21 genotypes form a special genetic lineage with a novel GBS that binds only Le^a^ antigen of HBGAs (22–24). These previous data raise the question of what the advantage of the new GBS with such narrow binding spectrum over the original GII GBS is. We provide phenotypic and structural data to demonstrate that this new GBS recognizes a group of glycans that share a terminal β-Gal. In addition, we discuss the potential mechanism of the low prevalence of the GII.13/21 lineage in humans.

## RESULTS

### Glycan binding specificity of GII.13 P domain proteins.

**FIG 1:**
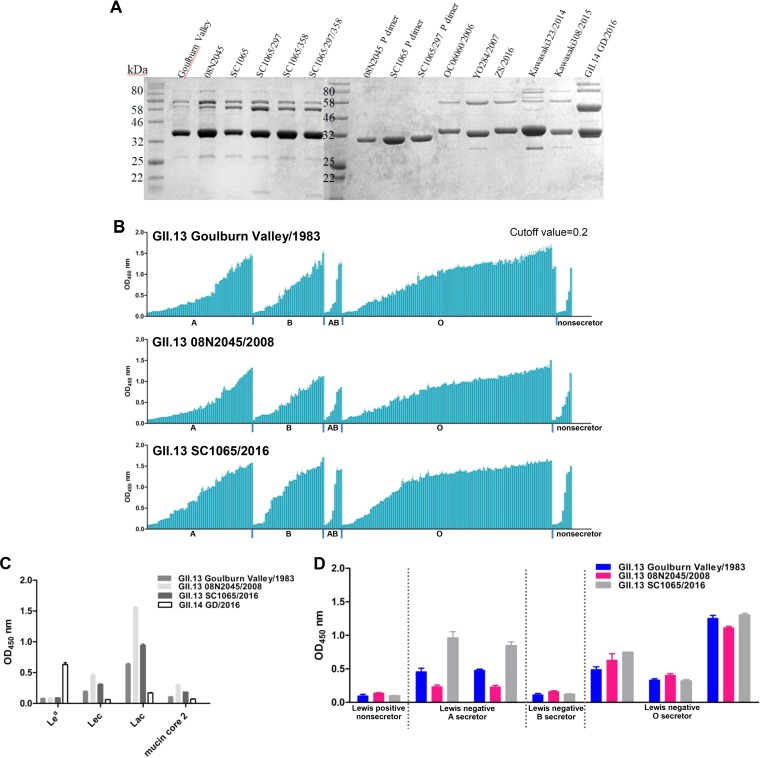
Saliva and glycan binding specificities of three GII.13 P proteins (Goulburn Valley/1983, 08N2045/2008, and SC1065/2016). (A) SDS-PAGE analysis of the P domain proteins of three GII.13 viruses, three GII.13 mutants, three GII.21 viruses, two GII.17 viruses, and a GII.14 virus. (B) Saliva-based binding assay showing binding signals (*y* axis) of the GII.13 proteins (0.5 μg/well) to type secretor samples (A, B, and O) and nonsecretor saliva samples (*x* axis). (C) Oligosaccharide-based binding assay showing that NoV GII.13 P proteins bind various synthetic polyacrylamide (PAA)-biotin-conjugated glycans in phosphate-buffered saline (PBS; pH 7.4). (D) Saliva-based binding assay of three GII.13 proteins to Lewis-positive nonsecretor samples and three kinds of Lewis-negative secretor samples, including A, B, and O. The experiment was repeated twice independently, and data are means and SDs (error bars) for absorbance values. OD_450 nm_, optical density at 450 nm.

Using saliva- and oligosaccharide-based binding assays, we determined the glycan binding specificities of recombinant P proteins of three GII.13 NoVs isolated at different times: Goulburn Valley/1983, 08N2045/2008, and SC1065/2016 (Fig. 1A). All three GII.13 P proteins exhibited broad-spectrum binding to all saliva samples representing type A, B, and O secretors and nonsecretors (Fig. 1B). Oligosaccharide-binding assays showed that these GII.13 P proteins bound to Galβ1-3GlcNAc (Lec) and Lac (Galβ1-4Glc). 08N2045/2008 and SC1065/2016 also bound weakly to mucin core 2 [Galβ1-3(GlcNAcβ1-6)GalNAc] (Fig. 1B). Furthermore, GII.14 GZ/2016 P protein, which has been shown to bind Lewis a antigen, served as a positive control (Fig. 1C). This is the first study to show that huNoV P domains bind mucin core oligosaccharides. We noted that these glycans share a terminal β-Gal, which may play a critical role in the binding outcomes. GII.13 and GII.21 P domains did not show obvious binding signals to Le^a^ antigen in our glycan binding experiments. In the saliva-based binding assays using saliva samples representing various Lewis phenotypes, including Lewis-positive nonsecretor, Lewis-negative A secretor, Lewis-negative B secretor, and Lewis-negative O secretor, GII.13 P proteins did not show any binding signals to the Lewis-positive nonsecretor samples but showed binding signals to the Lewis-negative samples. This result indicated that binding of these GII.13 P proteins was not dependent upon the Le^a^ antigen (Fig. 1D).

### Crystal structure of the GII.13 P dimer-Lec/core 2 complex.

**FIG 2:**
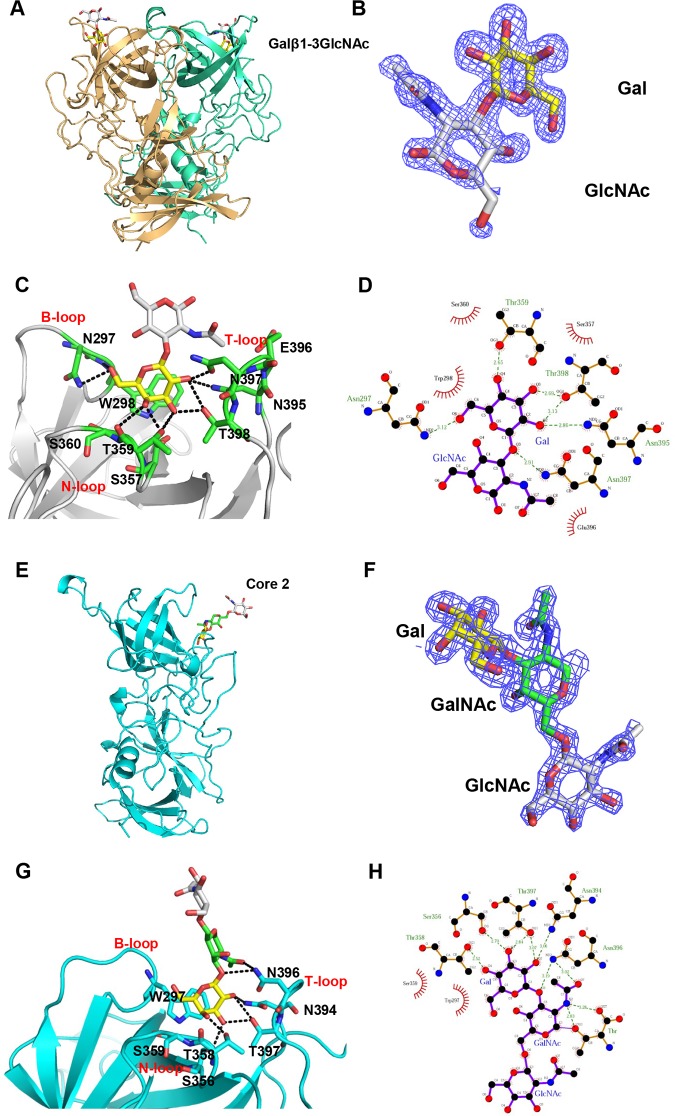
Crystal structures of GII.13 SC1065/2016 P dimer in complex with Lec disaccharide (A to D) and GII.13 08N2045/2008 P dimer in complex with mucin core 2 trisaccharide (E to H). (A and E) Cartoon representation of SC1065/2016 P dimer with two protomers (light orange and green/cyan) in complex with Lec disaccharide (Galβ1-3GlcNAc) and 08N2045/2008 P dimer with one protomer (cyan) in complex with mucin core 2 trisaccharide [Galβ1-3(GlcNAcβ1-6)GalNAc]. Lec disaccharide and mucin core 2 trisaccharide are shown in stick representation. Galactose (Gal), yellow; *N*-acetylglucosamine (GlcNAc), gray; *N*-acetylgalactosamine (GalNAc), green. The oxygen and nitrogen atoms of the disaccharide and trisaccharide are shown in red and blue, respectively. (B and F) (2mFo-DFc) omit electron density maps of the Lec disaccharide (B) and mucin core 2 trisaccharide (F) in GII.13 SC1065/2016 and GII.13 08N2045/2008 P protein structures, respectively. The mesh map of Lec (B) was contoured at 1σ and that of mucin core 2 (F) was contoured at 1.5σ (blue) around the selection site, with a coverage at a 1.6-Å radius. (C) Network of hydrogen bond interactions between SC1065/2016 and Lec disaccharide, represented by black dotted lines. The amino acids involved in the interaction are shown in stick representation in green. Gal, yellow; GlcNAc, gray. (D and H) Schematic diagram of the SC1065/2016-Lec disaccharide (D) and 08N2045/2008-mucin core 2 trisaccharide (H) interaction. The details of the interaction of SC1065/2016-Lec and 08N2045/2008-mucin core 2, mediated by hydrogen bonds and hydrophobic contacts, were analyzed using LIGPLOT. Hydrogen bonds, green dashed lines; hydrophobic contacts, red arcs. Black, red, and blue represent carbon, oxygen, and nitrogen atoms, respectively. (G) The hydrogen bonding network between 08N2045 and mucin core 2 trisaccharide is indicated by dashed lines. The amino acids participating in the interaction are shown in stick representation in cyan.

To further explore the structural basis of the observed GII.13 huNoV-glycan interaction, we solved the crystal structure of the GII.13 SC1065/2016 P domain in complex with Lec disaccharide to a 1.6-Å resolution in the P12_1_1 space group (Fig. 2A). Lec disaccharide was visible in the (2mFo-DFc) omit difference electron density map (Fig. 2B), and two sugar rings fitted into the map. The Lec-binding sites are located on the top of each P domain (Fig. 2A). Eight residues from the P2 domain were involved in binding to the β-Gal of Lec via hydrogen bonding and hydrophobic interactions (Fig. 2C). Specifically, W298 from the B loop, S357 from the N loop, and N395 and T398 from the T loop formed the bottom region of the binding pocket, and N297 from the B loop, T359 and S360 from the N loop, and N397 from the T loop constituted the edge region of the binding pocket. The β-Gal interacts with the side chains of N297, S357, T359, N395, and N397 through hydrogen bonds, while T398, S360, and W298 form hydrophobic interactions with β-Gal to support the binding outcomes (Fig. 2C and D). In contrast, the other saccharide of the Lec disaccharide, *N*-acetyl-β-glucosamine (β-GlcNAc), points away from the surface of the P dimer and as a result, only a hydrophobic interaction was formed with E396.

The crystal structure of the GII.13 08N2045/2008 P domain in complex with mucin core 2 trisaccharide was also determined at a 1.7-Å resolution (Fig. 2E). All three sugar rings were evident in the electron density map (Fig. 2F). Similar to Lec, mucin core 2 interacted with the GII.13 P domain mainly through the β-Gal. Seven residues were involved in this interaction, including W297, S356, T358, S359, N394, N396, and T397 (Fig. 2G). Residues S356, T358, N394, N396, and T397 contributed to the major interactions by forming hydrogen bonds with β-Gal, while W297 and S359 were involved in hydrophobic interactions (Fig. 2H). In addition, the GalNAc formed a hydrogen bond with N396, while the GlcNAc of the mucin core 2 trisaccharide pointed away from the surface of the P dimer without directly participating in the interactions. These structural data show that the GII.13 P dimer interacts with Lec/core 2 glycans mainly by the common terminal β-Gal.

### Mutation study of the GII.13 GBS.

**FIG 3:**
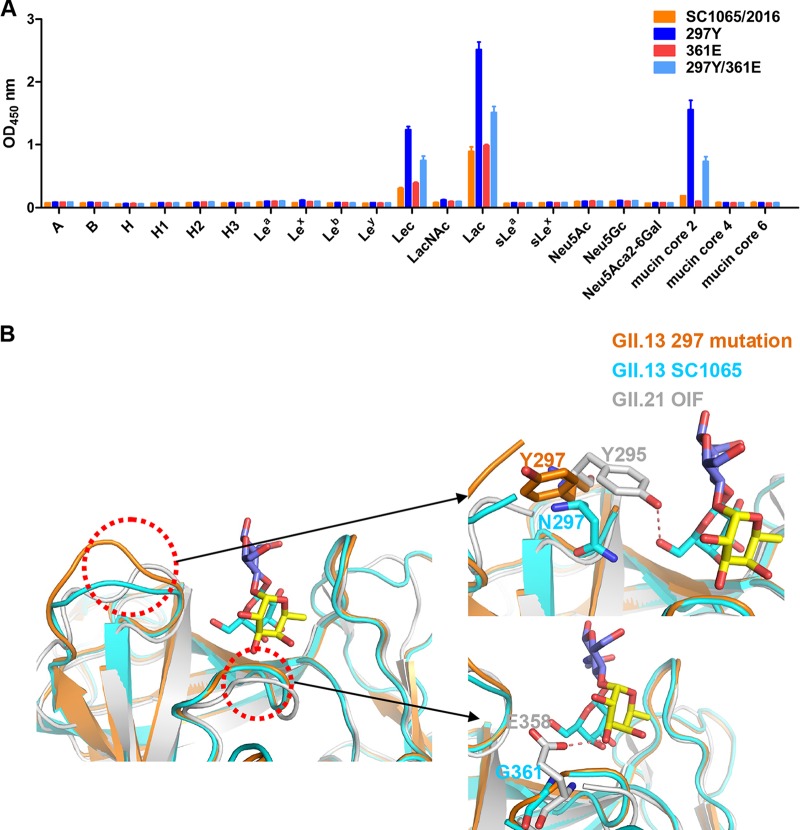
Glycan binding of the mutated GII.13 P proteins and GBS structural comparisons of GII.13/GII.21. (A) ELISA-based glycan binding assay of the 297Y and 361E and 297Y/361E mutations and native GII.13 SC1065/2016 P proteins. (B) Structure superposition of GII.13 SC1065 and GII.13 297 mutation based on GII.21 P dimer structure in complex with Le^a^ (PDB code 4RM0). Le^a^ is shown in stick representation. Gal, blue; GlcNAc, purpleblue; Fuc, yellow. The zoomed image shows structure superposition of two GII.13 proteins and GII.21 OIF as a cartoon representation. The P dimer interacts with Le^a^ in the upper right image, and the bottom right is an enlargement at GII.13 297Y/N/GII.21 295Y and GII.13 G361/GII.21 E358 shown in stick representation, respectively. Hydrogen bond interaction between 295Y of GII.21 OIF and Gal or E358 and α-Fuc is represented by red dashed lines.

Although the GII.13 and GII.21 OIF GBSs share high genetic and structural similarity, we noted two amino acid mutations: the residue at 297 in the GII.13 SC1065 was an asparagine (N), but it was a tyrosine (Y) in GII.21 OIF, while G361 in the GII.13 SC1065 changed to an E358 in GII.21 OIF. Because the GII.21 OIF P domain was reported to bind Le^a^ antigen (23) but the GII.13 P domains in our study did not, three reverse mutants of the GII.13 (SC1065/2016) P domain were constructed to explore the role of these amino acids in glycan binding. These included a single mutation (N297Y or G361E) and a double mutation (N297Y/G361E) (Fig. 1A), aiming to restore the Le^a^ binding function. Unexpectedly, oligosaccharide-based binding assays showed that all mutant P domains retained their original pattern of binding to Lec/lac/mucin core 2 glycans and did not bind to Le^a^ antigen (Fig. 3A). Also, the single mutant N297Y and the double mutant N297Y/G361E showed stronger binding signals to all Lec/Lac/mucin core 2 glycans and particularly to mucin core 2, while E361 showed the same binding affinity for the glycans as the native P proteins (Fig. 3A).

We determined the crystal structure of the SC1065/2016 N297Y mutant. Further structural comparison showed that all other residues involved in glycan binding were conserved among GII.13/21 NoVs, with an exception of a change from Y295 in GII.21 OIF to N297 in GII.13 (Fig. 3B), likely presenting different orientations. Y297 in the crystal structure of an SC1065 N297Y mutant displayed an orientation opposite that in GII.21 OIF (Fig. 3B). The residue E358, which forms a hydrogen bond with the fucose of Le^a^ in GII.21 OIF (23), changed to G361 in GII.13 (Fig. 3B), likely losing the potential interaction. These differences of amino acid conformation may affect the binding specificity and/or affinity of the P protein with the glycan ligands, which may contribute to the lack of binding to the Le^a^ antigen of GII.13 in this study.

### GII.21 glycan binding specificity.

**FIG 4:**
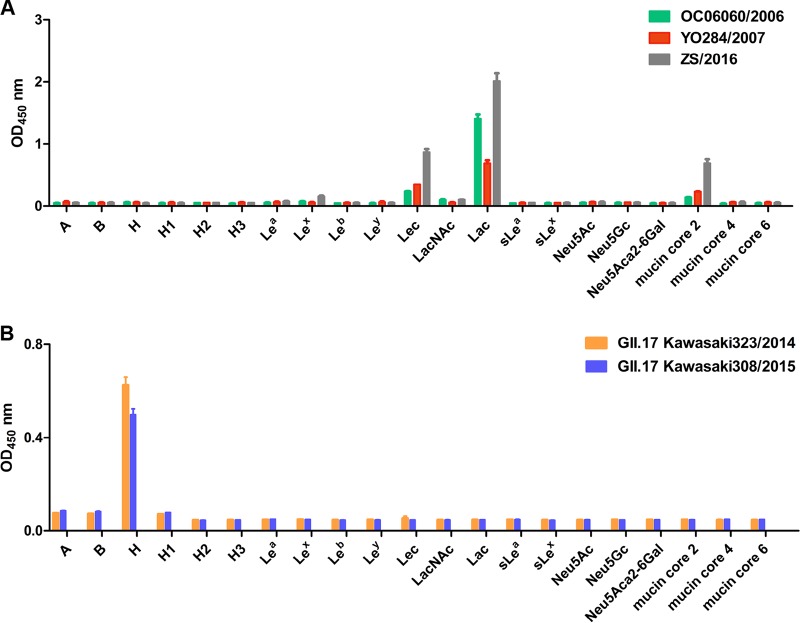
Glycan binding specificity of GII.21 and GII.17 P proteins for oligosaccharides. (A) ELISA-based assay of three GII.21 (OC06060/2006, YO284/2007, and ZS/2016) P proteins binding to oligosaccharides. (B) ELISA-based assay of two GII.17 (Kawasaki323/2014 and Kawasaki308/2015) P proteins to oligosaccharides. The experiment was repeated three times independently, and data are means and SDs (error bars).

The different glycan binding profiles of the three GII.13 NoVs in this study and the previously reported GII.13 VP1 (GenBank accession number BAQ94583 [22]) and GII.21 OIF (23) prompted us to investigate the glycan binding patterns of other GII.21 strains. To this end, we produced P domain proteins of three GII.21 strains (OC06060/2006, YO284/2007, ZS/2016) and those of two GII.17 strains (Kawasaki323/2014, Kawasaki308/2015) that were closely related to the GII.13/21 lineage as controls (Fig. 1A). Oligosaccharide-based binding assays showed that all three GII.21 P proteins bound to Lec, Lac, and mucin core 2 glycans, a binding profile similar to that of the GII.13 NoVs (Fig. 4A). In contrast, two GII.17 P proteins bound to type H disaccharides (Fuca1-2Gal), consistent with the previously reported binding between GII.17 Kawasaki308 P domain and 2′FL (Fucα1-2Galβ1-4Glc) (15) (Fig. 4B). Thus, GII.13 and GII.21 P proteins showed similar binding specificities in this study.

### Inhibition of glycan and saliva binding of GII.13/21 P domains by nonfat milk and lactose.

**FIG 5:**
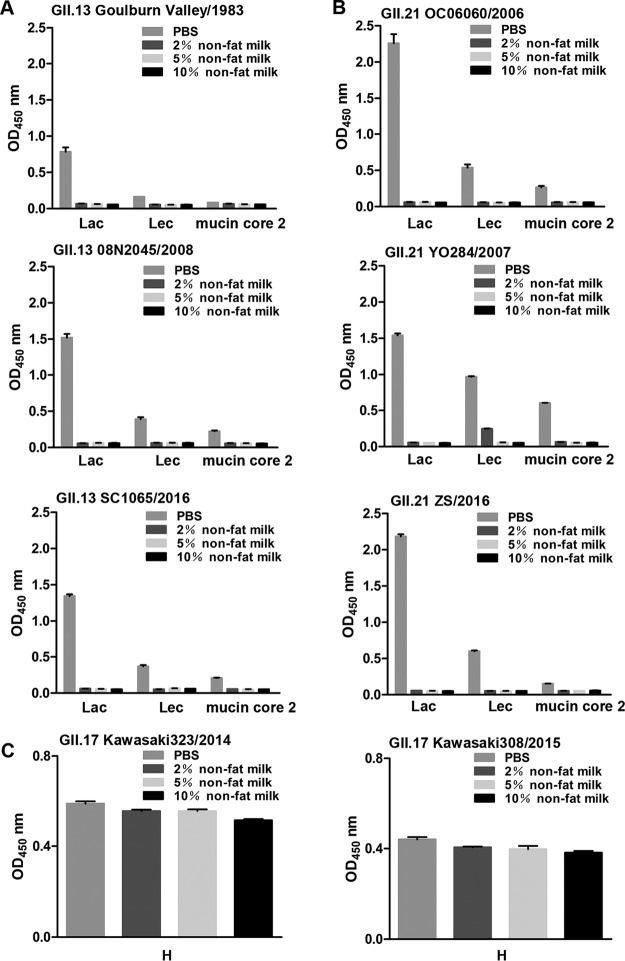
Inhibition of the binding of GII.13/21/17 P proteins to glycans by nonfat milk. (A) Blocking by four concentrations (0%, 2%, 5%, and 10%) of nonfat milk of P proteins of GII.13 Goulburn Valley/1983, 08N2045/2008, and SC1065/2016 binding to Lec, Lac, or mucin core 2. The *x* axis shows synthetic PAA-conjugated Lec, Lac, or mucin core 2. (B) Blocking by four concentrations (0%, 2%, 5%, and 10%) of nonfat milk of P proteins of GII.21 OC06060/2006, YO284/2007, and ZS/2016 binding to Lec, Lac, or mucin core 2. (C) Binding of GII.17 Kawasaki323/2014 and Kawasaki308/2015 P proteins to blood type H disaccharide mixed with nonfat milk (0 to 10%).

**FIG 6:**
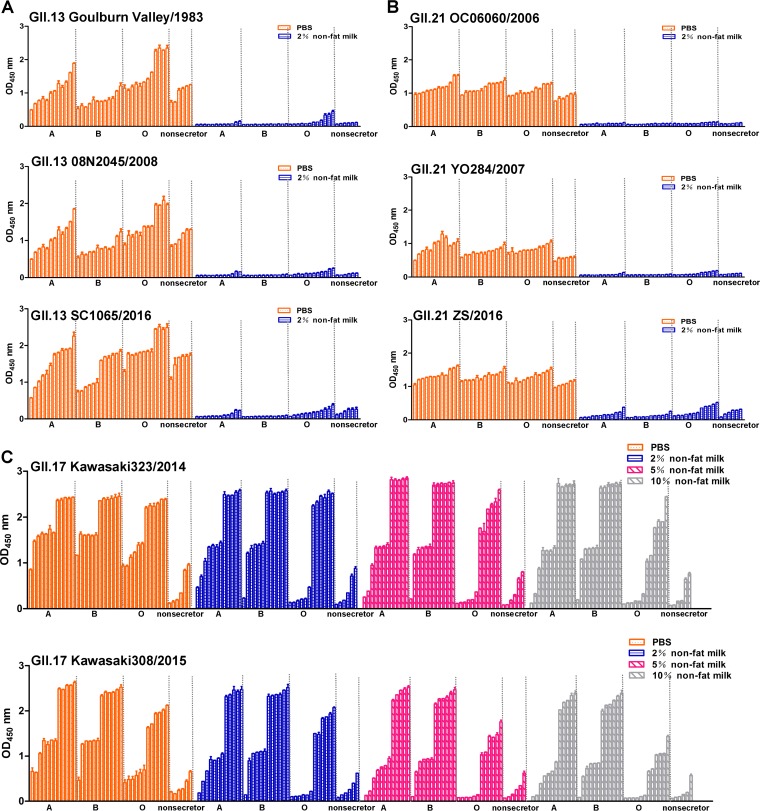
Nonfat milk blocked the binding of GII.13/21/17 P proteins to saliva. (A) Two concentrations (0% and 2%) of nonfat milk blocked the binding of the P proteins of GII.13 Goulburn Valley/1983, 08N2045/2008, and SC1065/2016 to secretor (A, B, and O), and nonsecretor saliva. (B) Two concentrations (0% and 2%) of nonfat milk blocked the binding of the P proteins of GII.21 OC06060/2006, YO284/2007, and ZS/2016 to secretor (A, B, and O) and nonsecretor saliva. (C) Binding of GII.17 Kawasaki323/2014 and Kawasaki308/2015 P proteins mixed with nonfat milk (0 to 10%) to type secretor (A, B, and O) and nonsecretor saliva.

Consistent with the binding to lactose, nonfat milk blocked the glycan binding function of the GII.13 and GII.21 P proteins (Fig. 5A and B) but did not affect the HBGA binding of the GII.17 P protein (Fig. 5C). Two percent nonfat milk blocked the binding of GII.13/21 P protein to saliva (Fig. 6A and B). However, even high-concentration (10%) nonfat milk cannot inhibit the binding of GII.17 P protein to saliva (Fig. 6C). These data suggest that some components of nonfat milk occupy the GBS of the GII.13/GII.21 P domain and thus inhibit binding to Lac/Lec/mucin core 2 glycans and saliva.

**FIG 7:**
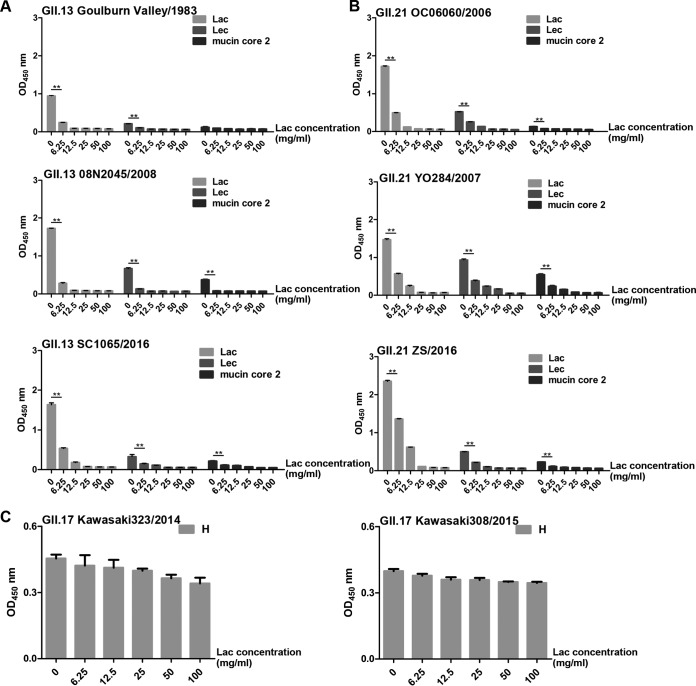
Inhibition of P protein-glycan interactions by free lactose. (A) Six concentrations of free lactose blocked binding of the GII.13 Goulburn Valley/1983, 08N2045/2008, and SC1065/2016 P proteins to synthetic PAA-conjugated Lec and mucin core 2 glycans. Synthetic PAA-conjugated Lac was included as a positive control. (B) Six concentrations of free lactose blocked binding of the GII.21 OC06060/2006, YO284/2007, and ZS/2016 P proteins to synthetic PAA-conjugated Lac, Lec, and mucin core 2 glycans. (C) Binding of the GII.17 Kawakasi323/2014 and Kawakasi308/2015 P proteins to blood type H disaccharide mixed with six concentrations of free lactose. The *x* axis shows the concentrations of free lactose. The experiment was repeated three times independently, and data are means and SDs (error bars).

**FIG 8:**
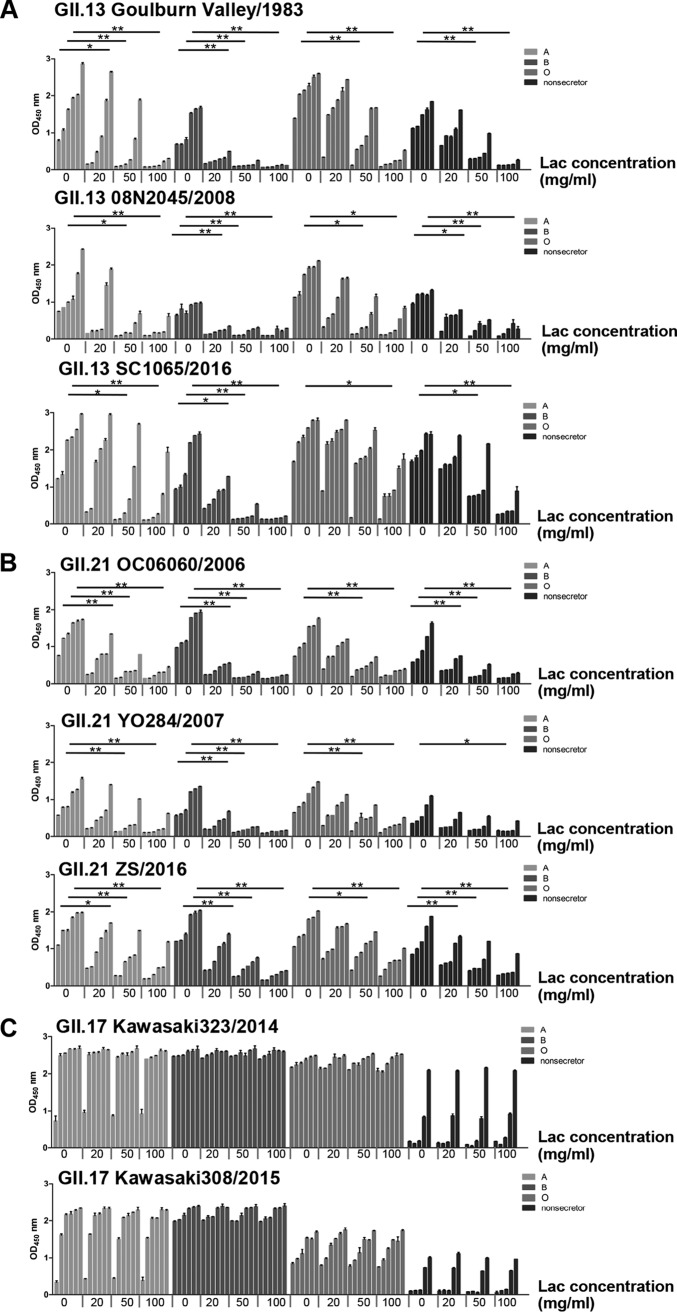
Inhibition by free lactose of the binding of P proteins to saliva. (A) Four concentrations of free lactose blocked the binding of P proteins of GII.13 Goulburn Valley/1983, 08N2045/2008, and SC1065/2016 to secretor (A, B, and O) and nonsecretor saliva. (B) Four concentrations of free lactose blocked the binding of P proteins of GII.21 OC06060/2006, YO284/2007, and ZS/2016 to secretor (A, B, and O) and nonsecretor saliva. (C) Binding of P proteins of GII.17 Kawakasi323/2014 and Kawakasi308/2015 to type secretor (A, B, and O) and nonsecretor saliva. The *x* axis shows the free-lactose concentrations. The experiment was repeated three times independently, and data are means and SDs (error bars).

Similarly, a lactose blocking assay showed that free Lac oligosaccharides blocked binding of the GII.13 and GII.21 P domain proteins to Lec, mucin core 2-PAA conjugate (Fig. 7A and B), and saliva (Fig. 8A and B), but not the binding of GII.17 P domain protein to H antigen or saliva (Fig. 7C and 8C). Therefore, lactose is an effective blocking reagent for the observed GII.13 and GII.21 P domain-glycan binding.

## DISCUSSION

The GII.13/21 genetic lineage gains a novel GBS distinct from those of the other GII NoVs. However, the questions of why, how, and when this GII.13/21 lineage emerged from mainstream GII NoVs, most likely a GII.17 NoV (22), is unclear. A basic question is what glycans this novel GBS binds. Previous studies on this genetic lineage concluded that the GII.13/21 GBS binds to Le^a^ antigen, which is one of the many HBGAs (22, 23). However, such a narrow binding spectrum appears not to be sufficient, simply because it lacks the typical diversity nature of huNoVs and does not show an advantage of the new GBS over the original one. Part of this puzzle has been solved by this study, as we have offered solid evidence to demonstrate that the GII.13/21 GBS recognizes a group of glycans that share a terminal β-Gal, including Lec, Lac, and mucin core 2. Importantly, the crystal structures of the GII.13 P domains in complex with Lec and mucin core 2 suggest that the interaction of the GII.13/21 GBS with these glycans is mediated by β-Gal. These bindings occur without participation of a Fuc, indicating a binding mode distinct from those of GI and the mainstream GII huNoVs (4–6).

**FIG 9:**
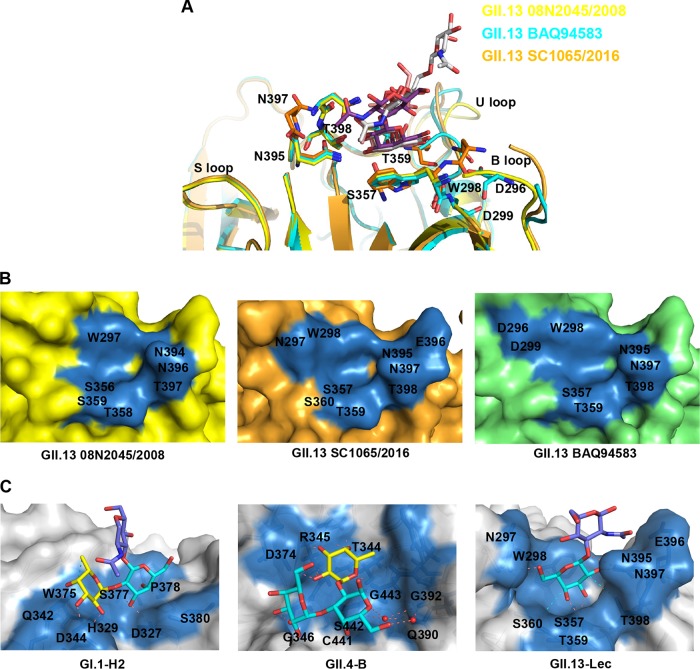
Structural comparison of GII.13 in complex with Le^a^, Lec, and mucin core 2 and models of NoV-glycan ligand interactions. (A) Structural superimposition of GII.13 VP1 (GenBank accession number BAQ94583) and SC1065/2016 08N2045/2008 in complex with Le^a^, Lec, and mucin core 2. Residues in the glycan binding site of GII.13 SC1065, GII.13 08N2045, and GII.13 VP1 are shown in stick representation in orange, yellow, and cyan. Amino acids residues of GII.13 VP1 in glycan binding site are in black. Le^a^, Lec, and mucin core 2 are shown in stick representation in pink, purple, silver, respectively. (B) Surface representations of GII.13 08N2045/2008, VP1, and SC1065/2016. GII.13 08N2045/2008, yellow; VP1, green; SC1065/2016, orange. The binding pocket in the GII.13 08N2045/2008, VP1, and SC1065/2016 glycan binding site is in blue, and the amino acid residues that interact with mucin core 2, Lec, and Le^a^ are indicated. (C) Interaction networks between NoVs and glycan ligands: GI.1 (Norwalk virus)-H2 (PDB code 2ZL6), GII.4 (NSW0514)-B (PDB code 4OP7), and GII.13 (SC1065)-Lec. Residues in the glycan binding site of NoVs are shown in surface representation in cyan. The amino acid residues that interact with glycans are indicated in black. Hydrogen bonds, deep salmon dashed lines; hydrophobic contacts, cyan dashed lines; water-mediated interactions, a red ball. The glycans of H2, B, and Lec are shown as sticks. Fuc, yellow; Gal, cyan; GlcNAc, purple.

Although the GII.13/21 GBS appears to rely mainly on the terminal β-Gal, some tested glycans with such a terminal β-Gal did not bind the GII.13/21 P domains. For example, the P domains of the selected GII.13/21 NoVs bound Lec, lactose, and mucin core 2 but did not bind Le^a^ or Le^x^, which contains a terminal β-Gal. To investigate this, we performed a structural superimposition of the three P domains in complex with Le^a^, Lec, and mucin core 2 (Fig. 9A). This structural comparison showed that the GBSs were highly conserved but did not indicate a clash of the three glycans with any part of the GBSs (Fig. 9B). Also, an attempt to shift the binding of GII.13 SC1065/2016 to Le^a^ by reverse mutation of residues 297 and/or 361 failed. Thus, why the GII.13/21 P domain binds some β-Gal-containing glycans but not others, as well as the reason for the various binding strengths, is unclear.

The glycan binding profiles of huNoVs have been shown to be associated with their infection risk (3, 24–29), and possibly also their prevalence (19, 21). We showed that the GII.13/21 huNoVs with the new GBS recognize a broad spectrum of terminal β-Gal-containing glycans, including mucin core 2. Mucin core 2 polymerizes at the C and N termini of many proteins in gastrointestinal tract and these proteins with mucin core 2 were shown to be secreted to intestinal lumen, forming a gel-like network as the main structure of mucus (30). This mucin core 2-containing mucus may act as an attachment factor for GII.13/21 NoVs, similar to HBGAs for other huNoVs. However, except for a slight increase of GII.13 prevalence in Nepalese children between 2005 and 2011 (31), the worldwide prevalence of GII.13/21 NoVs appears not to be high according to previous reports (32, 33) and the NoroNet/CaliciNet public databases (34). This seems conflict with the fact that GII.13/21 P domains bound the majority of human saliva samples tested in this study, which are rich in β-Gal-containing glycans. While the distribution of β-Gal-containing glycans on the human intestinal mucosal epithelium is unknown, it is plausible to assume that many β-Gal-containing glycans are there, because β-Gal is a common component of mammalian glycans. So, why is the prevalence of GII.13/21 huNoVs low? One possibility is related to the β-Gal saccharide-binding mode of the GII.13/21 GBS, which may not bind the glycan attachment factor with high affinity. Although we detected binding by enzyme-linked immunosorbent assay (ELISA), the binding affinity between single-β-Gal- or terminal-β-Gal-containing glycans and the GII.13/21 GBS should be determined using methods with greater accuracy.

A more important factor contributing to the low prevalence of the GII.13/21 huNoVs may be the GBS of the GII.13/21 huNoVs, which is easily interfered with. The function of the GII.13/21 GBSs appears to be easily blocked or inhibited by low-level glycerol molecules (22, 23), suggesting that other small molecules with a glycine link structure also inhibit the GII.13/21 GBS. Indeed, we also detected a glycerol molecule in the GII.13 08N2045/2008 P dimer. Moreover, nonfat milk and lactose oligosaccharides inhibited the binding of GII.13/21 P proteins to their ligands. Because lactose and lactose-like molecules are abundant in milk, these molecules may be extensively present in the gastrointestinal tract of breastfeeding infants and milk-drinking adults. Thus, lactose and other free glycans containing terminal β-Gal molecules may function as decoy receptors (35) for GII.13/21 huNoVs, thus reducing the risk of infection. This may explain the low prevalence of GII.13/21 huNoVs. Based on this principle, compounds containing β-Gal glycans may function as antivirals against infection by GII.13/GII.21 huNoVs. Another potential significance for the emergence of the new GBS in GII huNoVs may be to escape herd immunity. By targeting individuals spared by the dominant GII strains, the new GBS might allow GII.13/GII21 strains to find a niche of susceptible individuals, even though it is quite limited. This may represent one of the potential NoV potentiality to reach their current diversity.

In conclusion, GII.13/21 huNoVs have developed a novel GBS that interacts with glycans via the common β-Gal through a unique glycan binding mode. This binding mode differs from that of the conventional GBS of the mainstream GII HuNoVs, which relies on the α-Fuc as the MaBS (Fig. 9C). Although GII.13/21 huNoVs exhibit wide glycan binding spectra that should facilitate their infection and prevalence, the decoy glycan receptors in nature may limit their prevalence. Our findings provide new insights into the host interaction, evolution, and epidemiology of huNoVs, which may facilitate development of strategies for control and prevention of huNoVs.

## MATERIALS AND METHODS

### Ethics statement.

The saliva samples for this research were approved by the meeting of ethics committee of the National Institute for Viral Disease Control and Prevention, China CDC (Beijing, China; protocol number IVDC2017NO.023). The saliva samples from 225 children were collected previously and stored in our laboratory. The children were between 3 and 8 years old, and the ratio of boys to girls was 1.5:1; some of these children suffered from NoV infections. Written informed consent was obtained from participants and the parents of all children who provided specimens. All samples were anonymized. The production of New Zealand rabbit antiserum was approved by the Animal Care Welfare Committee of National Institute for Viral Control and Prevention, China CDC (Beijing, China; protocol number 20160715023).

### P domain expression and purification.

The following P domain proteins were produced and tested in this study: (i) GII.13 P domain protein SC1065/2016 (accession number MK408529; all accession numbers listed are GenBank accession numbers) isolated from a sporadic case of GII.13 NoV in 2016 in Sichuan, China, (ii) two GII.13 P domains based on the sequences of Goulburn Valley/1983 (DQ379714) and 08N2045/2008 (AB809973), (iii) three GII.21 P domains based on the sequences of OC06060/2006 (AB542916), YO284/2007 (KJ196284), and ZS/2016 (KY407202), (iv) two GII.17 P domains based on the sequences of the outbreak isolates Kawasaki323/2014 (AB983218) and Kawasaki308/2015 (LC037415), and (v) a GII.14 P domain protein GD/2016 (MK850443) isolated from a sporadic case of GII.14 NoV in 2016 in Guangdong, China. The P domain-encoding sequences were chemically synthesized by Genewiz Company (Suzhou, China). The three GII.13 P domain (SC1065) mutants with 297Y or 361E single mutations, or with the 297Y/361E double mutation, were constructed by overlap PCR using the mutation sites in the corresponding primer pairs.

Except for the GII.14 GD/2016 P protein, all P domains were cloned into the pGEX-6P-1 vector. For P protein production, we added a cysteine-containing short peptide (CDCRGDCFC) to the C-terminal end of the P domain to stabilize P protein formation (5), while for P dimer production, the wild-type P domain sequences were used (36). The GII.14 GD/2016 was cloned into a modified His-tagged pET-28a-SUMO vector and expressed with a SUMO fusion protein; the SUMO protein did not bind to HBGAs. The His-tagged fusion P protein was purified using a HiTrap Fast Flow (GE Healthcare Life Sciences), digested with PreScission protease overnight at 4°C as previously described (37). Other the P domains expressed with a glutathione *S*-transferase (GST) fusion protein, as described previously (38). The P proteins and dimers were purified using glutathione-Sepharose 4B (GE Healthcare Life Sciences), the GST tag was cleaved with PreScission protease, and the P dimer was further purified by Superdex 200^16/600^GL gel filtration chromatography using phosphate-buffered saline (PBS; 140 mM NaCl, 2.7 mM KCl, 10 mM Na_2_HPO_4_, 1.8 mM KH_2_PO_4_ [pH 7.4]). P proteins contained 12 P dimers (39).

### Glycan binding and blocking assays.

The binding assays of biotin-labeled polyacrylamide (PAA)-conjugated oligosaccharide and P proteins were confirmed by enzyme-linked immunosorbent assay (ELISA), as described previously (40). The P proteins at 20 μg/well were used to coat microtiter plates at 4°C overnight. After blocking with 5% nonfat milk at 37°C for 2 h, the following synthetic oligosaccharides at 0.2 μg/well were incubated at 4°C overnight in PBS: blood type A and B trisaccharides (A and B), H disaccharide (H), H type 1 (H1), H type 2 (H2), H type 3 (H3), Lewis a (Le^a^), Lewis x (Le^x^), Lewis b (Le^b^), Lewis y (Le^y^), type I precursor (Lec), type II precursor (LacNAc), lactose (Lac), sialyl Le^a^ (sLe^a^), sialyl Le^x^ (sLe^x^), Neu5Ac, Neu5Gc, Neu5Acα2-6Gal, and mucin cores 2, 4, and 6 (GlycoTech); text formulas and catalogue numbers are listed in Table 1. The plates were incubated with horseradish peroxidase (HRP)-conjugated streptavidin (Abcam) at 37°C for 1 h. Color was developed using the 3,3′,5,5′-tetramethylbenzidine (TMB) kit (BD Biosciences); the reaction was stopped by addition of 1 M phosphoric acid, and the absorbance at 450 nm was measured.

**TABLE 1:** Details for glycans in this study^*a*^

Glycan	Text formula	Catalogue number
A	GalNAcα1-3(Fucα1-2)Galβ-PAA-biotin	01-032
B	Fucα1-2Galα1-3Galβ-PAA-biotin	01-033
H	Fucα1-2Galβ-PAA-biotin	01-019
H1	Fucα1-2Galβ1-3GlcNAcβ-PAA-biotin	01-037
H2	Fucα1-2Galβ1-4GlcNAcβ-PAA-biotin	01-034
H3	Fucα1-2Galβ1-3GalNAcα-PAA-biotin	01-060
Lewis a	Galβ1-3(Fucα1-4)GlcNAcβ-PAA-biotin	01-035
Lewis b	Fucα1-2Galβ1-3(Fucα1-4)GlcNAcβ-PAA-biotin	01-042
Lewis x	Galβ1-4(Fucα1-3)GlcNAcβ1-3Galβ-PAA-biotin	01-036
Lewis y	Fucα1-2Galβ1-4(Fucα1-3)GlcNAcβ-PAA-biotin	01-043
LacNAc	Galβ1-4GlcNAcβ-PAA-biotin	01-022
Lec	Galβ1-3GlcNAcβ-PAA-biotin	01-020
Lac	Galβ1-4Glcβ-PAA-biotin	01-021
Sialyl Le^a^	Neu5Acα2-3Galβ1-3(Fucα1-4)GlcNAcβ-PAA-biotin	01-044
Sialyl Le^x^	Neu5Acα2-3Galβ1-4(Fucα1-3)GlcNAcβ1-3Galβ-PAA-biotin	01-045
Neu5Ac	α-Neu5Ac-PAA-biotin	01-012
Neu5Gc	Neu5Gcα-PAA-biotin	01-051
Neu5Acα2-6Gal	Neu5Acα2-6Galβ-PAA-biotin	01-105
Mucin core 2	Galβ1-3(GlcNAcβ1-6)GalNAcα-PAA-biotin	01-083
Mucin core 4	GlcNAcβ1-3(GlcNAcβ1-6)GalNAcα-PAA-biotin	01-089
Mucin core 6	GlcNAcβ1-6GalNAcα-PAA-biotin	01-073

a These glycans were purchased from GlycoTech.

Nonfat milk and free lactose (Sigma) were used in blocking assays to block glycan binding to Goulburn Valley, 08N2045, and SC1065 GII.13 P proteins, OC06060/2006, YO284/2007, and ZS/2016 GII.21 P proteins, and Kawasaki323/2014 and Kawasaki308/2015 GII.17 P proteins. In the nonfat milk blocking assay, Lec, Lac, or mucin core 2 at 0.2 μg/well was incubated at 4°C overnight with 0%, 2%, 5%, or 10% nonfat milk, while in the lactose blocking assay, Lec, Lac, or mucin core 2 was mixed with 0.625, 1.25., 2.5, 5.0, or 10.0 mg/well of free Lac in PBS. HRP-conjugated streptavidin was added, and the assay was completed as described above (40).

### Saliva binding and blocking assay.

A panel of 214 saliva samples with A, B, HI, Le^a^, Le^b^, Le^x^, and Le^y^ phenotypes was used in the saliva binding assay, as described previously (18, 41). Microtiter plates were coated with boiled saliva samples (1:1,000) in PBS at 4°C overnight. After 2 h of blocking with 5% nonfat milk, the P protein of GII.13 Goulburn Valley, 08N2045, and SC1065 was added at 0.5 μg/well and incubated at 4°C overnight. A rabbit anti-P domain SC1065 antibody diluted 1:6,000 and an HRP-conjugated goat anti-rabbit antibody (1:10,000) were used as the primary and secondary antibodies, respectively.

In nonfat milk and lactose blocking saliva assays, the saliva coating and nonfat milk blocking steps were performed as described above. For nonfat milk blocking and lactose blocking saliva assays, eight P proteins were added at 0.5 μg/well to 2%, 5%, or 10% skimmed milk powder and 2, 5, or 10 mg/well of free lactose, respectively. Anti-SC1065 (1:6,000) and anti-GII.17 KW323 (1:8,000) (18) primary antibodies were used in the assays of GII.13/GII.21 and GII.17 P proteins, respectively. Other steps were as described above (41).

### Protein crystallization.

The purified P domain of SC1065 and 08N2045 was concentrated to ∼10 mg/ml. Native crystals of the SC1065 and 08N2045 P proteins were grown using the sitting-drop vapor diffusion method by mixing 1 μl of protein solution with an equal volume of reservoir solution containing 0.2 M sodium citrate tribasic dehydrate, 20% (wt/vol) polyethylene glycol 3350, 0.2 M ammonium acetate, 0.1 M HEPES (pH 7.5), and 25% (wt/vol) polyethylene glycol 3350. SC1065 P protein and Lec disaccharide (Dextra) were mixed at a 1:50 molar ratio and incubated for 5 h at 4°C under the same conditions as the native protein and also like the complex crystals of 08N2045 P domain and core 2 trisaccharide. SC1065N297Y was crystallized under the condition of 8% (vol/vol) Tacsimate (pH 8.0) and 20% (wt/vol) polyethylene glycol 3350. After incubation for 7 days at 18°C, the native and complex crystals were transferred to a cryoprotectant containing mother liquor and 20% (vol/vol) glycerol and subsequently flash-frozen in liquid nitrogen.

### Data collection and processing.

X-ray diffraction data were collected at Shanghai Synchrotron Radiation Facility (SSRF) BL19U and processed with HKL2000. Additional processing was performed using CCP4 software. The structure of the GII.13 SC1065 P domain was determined using the molecular replacement module of PHASER, with the GII.21 P structure (Protein Data Bank [PDB] code 4RLZ) as a search model. The model was further refined using phenix.refine (42) in PHENIX (43) with energy minimization, ADP refinement, and bulk solvent modeling. The stereochemical quality of the final model was assessed using MolProbity. Data collection and refinement statistics are summarized in Table 2. The structural analysis was performed using PyMOL software (https://pymol.org/2/). The representative GII.13 P structures were calculated with the align function in PyMOL.

**TABLE 2:** Crystallographic X-ray diffraction and refinement statistics

Parameter^*a*^	Value(s) for:			
	GII.13 SC1065-Lec	GII.13 08N2045-mucin core 2	GII.13 08N2045	GII.13 SC1065-297 mutant
Data collection				
Space group	P12_1_1	P2_1_2_1_2_1_	P12_1_1	P2_1_2_1_2
Cell dimensions				
*a*, *b*, *c* (Å)	69.936, 122.935, 83.28	60.728, 99.695, 221.824	60.142, 107.512, 99.538	87.642, 105.667, 138.196
α, β, γ (°)	90, 90.066, 90	90, 90, 90	90, 98.308, 90	90, 90, 90
Resolution (Å)	50.00–1.60 (1.66–1.60)	50.00–1.70 (1.76–1.70)	50.00–1.50 (1.55–1.50)	50.00–1.50 (1.55–1.50)
*R*_merge_ (%)^*b*^	0.098 (0.586)	0.120 (0.824)	0.069 (0.272)	0.112 (1.361)
*I*/σ*I*	18.333 (3.250)	19.652 (3.187)	25.444 (7.358)	22.114 (1.954)
Completeness (%)	92.44 (69.77)	96.20 (89.60)	98.88 (98.97)	98.94 (91.26)
Redundancy	6.8 (6.3)	11.6 (12.2)	7.0 (7.0)	11.3 (11.4)
Refinement				
Resolution (Å)	49.12–1.60	48.63–1.70	41.00–1.50	49.35–1.50
No. of reflections	171,050	143,820	197,031	201,845
*R*_work_/*R*_free_	0.1855/0.2052	0.1649/0.1876	0.1777/0.1928	0.1881/0.1989
No. of atoms				
Total	11,282	11,371	11,191	11,206
Protein	9,618	9,623	9,653	9,574
Ligand/ion	104	114	18	0
Water	1,560	1,634	1,520	1,632
*B*-factors (Å^2^)				
Protein	14.75	17.42	17.10	18.43
Water	23.71	27.49	28.97	27.73
Ligand/ion	33.91	25.65	15.70	
Root mean standard deviations				
Bond lengths (Å)	0.004	0.004	0.004	0.004
Bond angles (°)	1.05	1.01	0.71	0.70
Ramachandran plot				
Favored (%)	95.77	97.06	96.73	96.24
Allowed (%)	4.15	2.94	3.19	3.68
Disallowed (%)	0.08	0.00	0.08	0.08

a Values in parentheses are given for the highest-resolution shell.

b *R*_merge_ = Σhkl |*I*−|/ΣhklI, where *I* is the intensity of unique relfection hkl and is the average over symmetry-related observations of unique reflection hkl; hkl is the reflection index.

### Statistical analysis.

All data were analyzed using SPSS 20.0 software. Analysis of variance (ANOVA) was used to compare the differences between untreated and free-lac-treated groups. In figures, statistical significance is indicated as follows: *, *P* < 0.05, and **, *P* < 0.01.

### Data availability.

The structures of 08N2045, SC1065-lec, 08N2045-Core2, and SC1065-297Y P domains have been deposited under PDB codes 6JYR, 6JYN, 6JYS, and 6JYO, respectively.
